# iTRAQ-Based Quantitative Proteomic Analysis of the Initiation of Head Regeneration in Planarians

**DOI:** 10.1371/journal.pone.0132045

**Published:** 2015-07-01

**Authors:** Xiaofang Geng, Gaiping Wang, Yanli Qin, Xiayan Zang, Pengfei Li, Zhi Geng, Deming Xue, Zimei Dong, Kexue Ma, Guangwen Chen, Cunshuan Xu

**Affiliations:** 1 State Key Laboratory Cultivation Base for Cell Differentiation Regulation, Henan Normal University, Xinxiang, Henan Province, China; 2 Henan Engineering Laboratory for Bioengineering and Drug Development, Henan Normal University, Xinxiang, Henan Province, China; 3 College of Life Science, Henan Normal University, Xinxiang, Henan Province, China; Moffitt Cancer Center, UNITED STATES

## Abstract

The planarian *Dugesia japonica* has amazing ability to regenerate a head from the anterior ends of the amputated stump with maintenance of the original anterior-posterior polarity. Although planarians present an attractive system for molecular investigation of regeneration and research has focused on clarifying the molecular mechanism of regeneration initiation in planarians at transcriptional level, but the initiation mechanism of planarian head regeneration (PHR) remains unclear at the protein level. Here, a global analysis of proteome dynamics during the early stage of PHR was performed using isobaric tags for relative and absolute quantitation (iTRAQ)-based quantitative proteomics strategy, and our data are available *via* ProteomeXchange with identifier PXD002100. The results showed that 162 proteins were differentially expressed at 2 h and 6 h following amputation. Furthermore, the analysis of expression patterns and functional enrichment of the differentially expressed proteins showed that proteins involved in muscle contraction, oxidation reduction and protein synthesis were up-regulated in the initiation of PHR. Moreover, ingenuity pathway analysis showed that predominant signaling pathways such as ILK, calcium, EIF2 and mTOR signaling which were associated with cell migration, cell proliferation and protein synthesis were likely to be involved in the initiation of PHR. The results for the first time demonstrated that muscle contraction and ILK signaling might played important roles in the initiation of PHR at the global protein level. The findings of this research provide a molecular basis for further unraveling the mechanism of head regeneration initiation in planarians.

## Introduction

Planarians have an extraordinary regenerative ability through the activation of pluripotent stem cells (also called neoblasts) [1–3]. Strikingly, planarian can regenerate a head at the anterior-facing wounds within a week after amputation. This process includes several distinct events: closure of the wound, cell apoptosis, blastema formation, and blastema cell differentiation [4–7]. More specifically, wound healing is activated to cover the exposed tissues in the first 30 min–3 h after amputation. Subsequently, neoblasts at the wound site undergo an initial mitotic peak within the first 6 h, and proceed the migration towards the wound at about 18 h, and then a spatially restricted mitotic response at 48–72 h to promote blastema formation [5, 8, 9]. Conversely, cell apoptosis at the wound site is tightly coordinated with the proliferative reaction, so as to ensure the appropriate proportions in the regenerating planarian [7]. A study reported that wound closure in planarians after amputation was facilitated by muscle contraction [10], but the molecular mechanism that triggers wound closure is still unknown. In this study, we firstly focus on the mechanism of wound healing at the early stage of planarian head regeneration (PHR) after amputation at the proteome level.

Planarian regeneration is a complex process, regulated by various signaling pathways. For instance, extracellular signal-related kinase (ERK) signaling and c-Jun N-terminal kinase (JNK) signaling were highly activated at the wound site following amputation and were necessary for planarian regeneration by regulating the formation and differentiation of blastema [11, 12]. Furthermore, the regenerating tissues of the planarian re-establish proper anterior-posterior (AP) polarity involving canonical Wnt/β-catenin and Hedgehog (Hh) signaling [13–15], and proper dorsal-ventral (DV) polarity involving BMP signaling [16]. Recent studies revealed that Wnt/β-catenin and Hh signaling in planarians were necessary for posterior polarity (tail regeneration), and its inhibition resulted in head regeneration at the wound site [14, 15, 17–19]. A recent study also confirmed the notion that posterior β-catenin signaling interfered with head tissue differentiation in planarians through negatively modulating ERK signaling [20]. Although signaling pathways involving blastema formation and proper polarity have been extensively studied during the regeneration in planarians, those important pathways for priming head regeneration in planarians need to be further excavated. Studies have reported that integrin-linked kinase (ILK) signaling played important roles in wound healing, cell adhesion and migration, and tissue regeneration [21–23], but no research is performed on the role of ILK signaling in PHR as well as the expression changes of proteins associated with ILK signaling in PHR.

Previous researches have focused on the characterization of the regeneration transcriptome of the planarian *Schmidtea mediterrane* and *Dugesia japonica* [24–26]. However, it fails to reflect the influence of post-transcriptional modifications and protein interactions. Delightedly, isobaric tags for relative and absolute quantitation (iTRAQ), a powerful tool to detect the kinds and abundance of proteins, has been widely used in proteomics studies owing to its advantages over two-dimensional (2D) gel electrophoresis [27]. At present, a proteomics analysis of the normal and stem cell-depleted planarian *Schmidtea mediterrane*, has been performed [28–31], but an investigation of the potential proteome implicated in the initiation of head regeneration in the planarian *Dugesia japonica* remains unknown.

The aim of this study was to identify a wide range of proteins that are differentially regulated at the early stage of PHR. In this study, iTRAQ combined with LC-MS/MS was employed to quantitatively assess protein expression profiles in the regenerating head fragments at 0 h, 2 h and 6 h after amputation. We found that muscle contraction and ILK signaling might play vital roles in the initiation of PHR through functional enrichment and IPA analysis. The findings of this research provide a molecular basis for further unraveling the mechanism of head regeneration initiation in planarians.

## Materials and Methods

### Ethics Statement

Planarian samples were collected from the source of Tagang reservoir, Xinxiang, Henan, China. The samples were handled in strict accordance with the guidelines of the Animal Care and Use Committee of Henan Normal University. We confirm that the samples collected were not privately owned or protected in any way and that the field studies did not involve endangered or protected species.

### Model preparation of planarian head regeneration

Planarians used in this study belong to the asexual strain of the species *Dugesia japonica*, and were collected from Tagang reservoir, which is located at the geographic coordinates of longitude (E114° 01'13.48") and latitude (N35°36'4.45"). Animals were cultured in autoclaved tap water in the dark at 20°C. Planarians with a length of 8–10 mm were selected, and starved for at least 1 week prior to use in experiments. A total of 45 planarians mentioned above were randomly assigned to three groups with 15 planarians per group. For regeneration studies, planarians were amputated transversely anterior to the pharynx, and the anterior end was allowed to undergo head regeneration. The regenerating planarians were collected at 0 h, 2 h and 6 h after amputation, and stored at –80°C for further use.

### Protein extraction and iTRAQ labeling

Protein extraction was performed using a procedure described previously [32]. Frozen samples were ground in liquid nitrogen to a fine powder and suspended in lysis buffer (30 mM Tris, 7 M urea, 2 M thiourea, 4% CHAPS, 65 mM DTT). The suspension was vortexed at 4℃ for 1 h, and then centrifuged at 20,000 g for 1 h. The protein concentration of each sample was determined using a 2D Quantification kit (GE Healthcare, USA).

A total of 100 μg of each sample was denatured, reduced and alkylated as described in the iTRAQ protocol (Applied Biosystems, USA). Each sample was digested with 0.1 μg/μL trypsin solution at 37°C, overnight. The digested peptides were dried by vacuum centrifugation. Control (0 h), 2 h and 6 h samples were respectively labeled with 114, 115 and 116 iTRAQ tags according to the manufacturer’s protocol (Applied Biosystems, USA). The three samples were pooled and vacuum-dried.

### LC-MS/MS, protein identification and data analysis

The pooled sample was separated on the Poly-LC strong cation exchange column (4.6 x 100 mm) on a Nano HPLC System (GE Healthcare) according to the method described in detail previously [33]. In this study, a total of 30 fractions were collected over the gradient, but some were pooled to give a final total of 10 fractions that were desalted using a PepClean C-18 spin column (Sigma, USA), and dried by vacuum centrifugation. Subsequently, the fractionated samples were analyzed by LC-MS/MS based on Q-Exactive mass spectrometer (Thermo Fisher Scientific, Waltham, MA, USA) [33, 34].

For peptide data analysis, raw mass data were processed using Proteome Discover 1.4 software and searched against the SwissProt database (March 2013) from Uniprot website (http://www.uniprot.org) using Mascot 2.2 (Matrix Science, London, UK). The analysis and search parameters were set as: trypsin as the digestion enzyme with allowance for a maximum of two missed cleavage, Carbamidomethyl (C) and iTRAQplex modification (K and N-terminus) as a fixed modification, Oxidation (M) as a variable modifications, peptide mass tolerance of 20ppm, fragment mass tolerance of 0.1 Da. The mass spectrometry proteomics data have been deposited to the ProteomeXchange Consortium [35] *via* the PRIDE partner repository with the dataset identifier PXD002100.

In order to measure the false discovery rate (FDR), the peptide mass spectra datasets were used to search a decoy peptide database. The following filters were used in this study, peptide FDR≤0.01 and each protein with at least 2 unique peptides. Expression changes of the identified peptides in the regenerating head fragments were calculated in comparison with the control based on the iTRAQ reporter ion intensities. Based on relative quantification and statistical analysis, 1.4-fold change cutoff was selected to categorize proteins as significantly changed, that is, proteins with iTRAQ ratios > 1.4 were considered to be up-regulated, whereas those with iTRAQ ratios < 0.714 were considered to be down-regulated.

### Quantitative real-time PCR analysis of gene expression

In this study, several genes including *EIF4A1*, *EIF4A3*, *H2B*, *DDX*, *CTNNB*, *CAM*, *YBX*, *GST* were selected for quantitative real time RT-PCR (qRT-PCR) analysis. The primers were designed with Primer Express 2.0 software according to mRNA sequences of the 8 target genes and internal control *ACTB* and synthesized by Shanghai Generay Biotech Co., Ltd. The genes and their primer sequences used for qRT-PCR analysis were provided in S1 Table. RNA extraction, cDNA synthesis and qRT-PCR were carried out according to the method described previously [36]. The expression level of gene was calculated using 2^-ΔΔCt^ method. *ACTB* gene was used as the internal control and the experiments were performed three times. The statistical significance in expression was estimated by t-test, and p < 0.05 was considered to be significant.

### Bioinformatics analysis

In order to characterize the expression patterns of the proteins identified in our quantitative proteomics analysis, k-means clustering was utilized to classify the differentially expressed proteins, and Expression Analysis Systematic Explorer (EASE) software on DAVID website was applied to conduct functional enrichment analysis as described in detail previously [37, 38]. EASE analyses were conducted to identify GO categories that were over-represented according to a modified Fisher's exact test. In this study, the GO categories with *P*-value < 0.05 were considered to be significantly over-represented at the early stage of PHR.

In addition, the differentially expressed proteins were analyzed by Ingenuity Pathway Analysis (IPA) version 9.0 (Redwood City, CA, http://www.ingenuity.com) software for predominant canonical pathways [39]. Briefly, a dataset containing these proteins and corresponding extremum of expression values was firstly uploaded into “Dataset Files” of the IPA. Then IPA core analysis was performed. Canonical pathways obtained in this study were identified from the IPA library based on Fisher’s exact test. The significance of the association between the differentially expressed proteins and canonical pathways was measured as described in the literature [40].

## Results

### Quantitative proteomics analysis revealed tissue proteome alterations at the early stage of planarian head regeneration

To identify proteins associated with the initiation of PHR, iTRAQ was employed to assess proteome changes at 2 h and 6 h following amputation (n = 15), and Q-Exactive mass spectrometer was used to obtain better coverage of tissue proteome. The mass data was searched against the SwissProt database using Mascot 2.2 search engine. The peptide FDR≤0.01 and each protein with at least 2 unique peptides were utilized to filter out the data, and 1595 proteins were indentified (S2 Table). The protein mass distribution mainly concentrated in 10–100 kDa which made up 88.5% of the proteins. The proteins with 2–5 peptides, 6–10 peptides, and above 11 peptides comprised 890, 399 and 306, respectively. Protein sequence coverage with 50–100%, 40–50%,30–40%, 20–30%, 10–20% and under 10% variation accounted for 10.85%, 11.85%, 15.67%, 20.56%, 23.51% and 17.56% coverage, respectively.

Next, we applied a threshold of >1.4-fold to identify proteins that were differentially expressed. A total of 162 proteins meeting the screening criteria were classified as differentially expressed. Among these proteins, 83 proteins were up-regulated and 79 proteins were down-regulated at 2 h and 6 h after amputation compared to control group (S3 Table).

### Validation of gene expression by qRT-PCR

**Fig 1:**
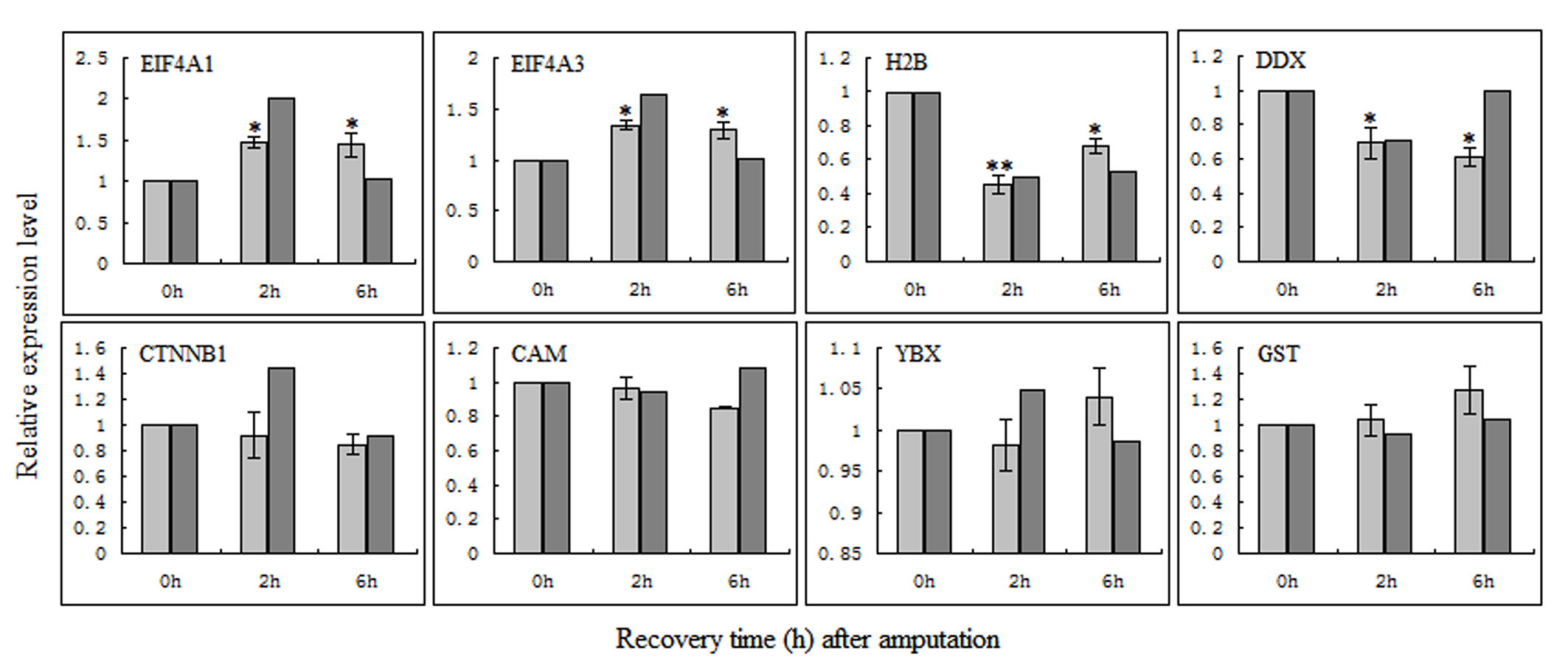
Validation of the identified proteins at the mRNA level by qRT-PCR analysis. Light gray histogram indicates mRNA levels detected by qRT-PCR; dark gray histogram indicates protein levels detected by iTRAQ. The mean values are represented in the bar graph. Bars represent standard errors (n = 3); * p<0.05.

Changes in the protein expression may be due to changes in its mRNA level. In this study, to investigate whether the changes observed in protein expression were the result of transcriptional regulation, we used qRT-PCR to determine mRNA levels of eight selected proteins (EIF4A1, EIF4A3, H2B, DDX, CTNNB1, CAM, YBX, and GST) in the regenerating head fragments at 0 h, 2 h and 6 h after amputation. As shown in Fig 1, in addition to CTNNB1 whose expression trend in protein level is not consistent with that in mRNA level, the changes in mRNA expression of the other seven proteins were basically correlated with their protein expression changes detected by iTRAQ approach. Furthermore, four proteins (EIF4A1, EIF4A3, H2B and DDX) had statistically significant differences, and three proteins (CAM, YBX and GST) had no statistically significant differences, which is consistent with the results from the proteomic analysis.

### Functional enrichment analysis of the differentially expressed proteins at the early stage of planarian head regeneration

**Fig 2:**
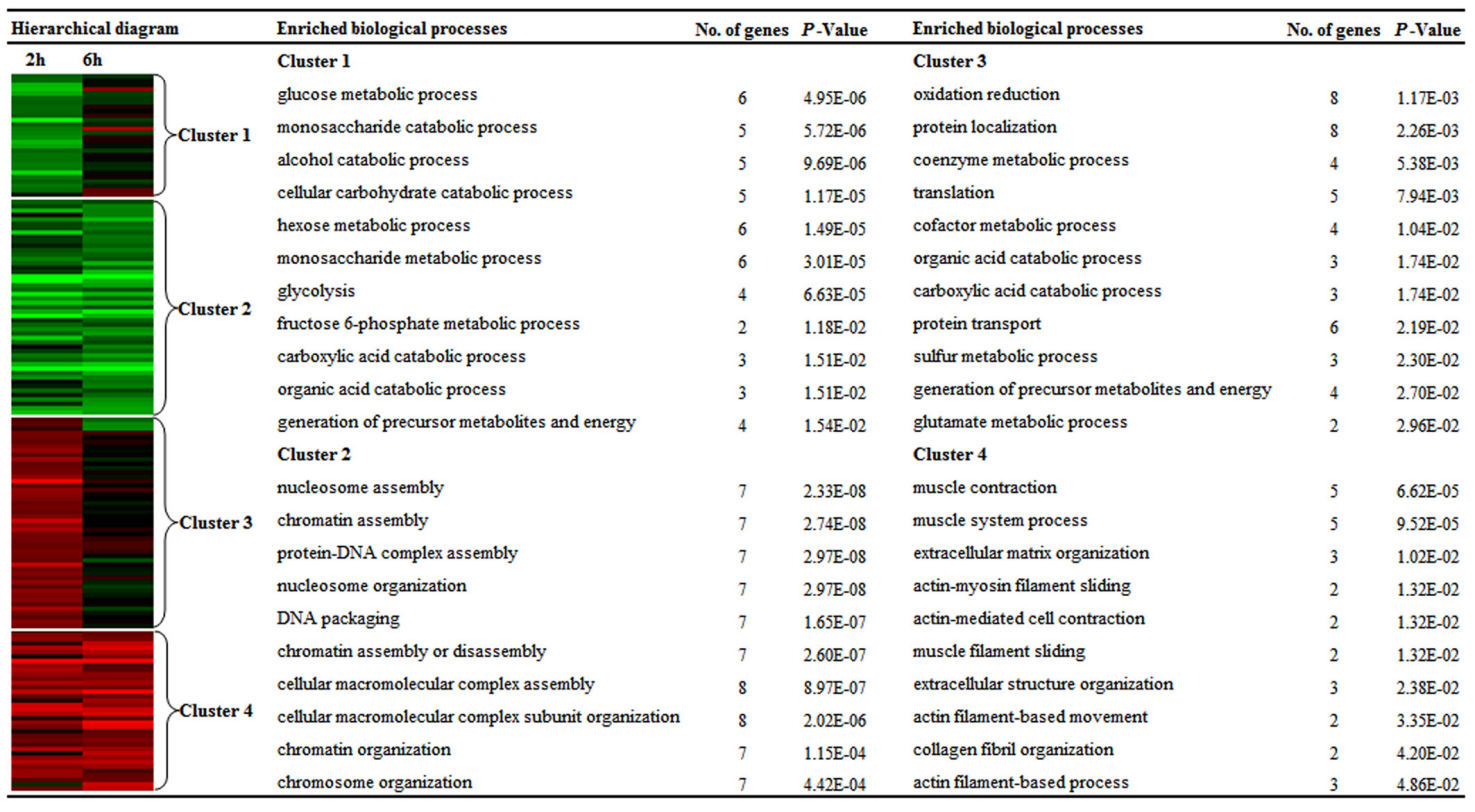
Global protein expression patterns at the early stage of planarian head regeneration. The “Hierarchical diagram” column represents K-means clustering of total of 162 differentially expressed proteins. Red and green colors denote the expression level higher and lower than the control, respectively. The over-represented functional categories of the four clusters were shown in the “Enriched biological processes” column. Categorical data were analyzed using a modified Fisher's exact test. *p* < 0.05 was considered to be significant.

In order to characterize the expression patterns of proteins that exhibited significant change at the early stage of PHR, k-means clustering was utilized to classify the differentially expressed proteins. The results showed that 162 proteins were categorized into 4 clusters: 28 proteins in cluster 1 and 50 proteins in cluster 2 were mainly down-regulated at 2 h and 6 h during PHR, while 48 proteins in cluster 3 and 36 proteins in cluster 4 were primarily up-regulated at 2 h and 6 h during PHR (Fig 2). Details for 162 differentially expressed proteins that grouped into four clusters were shown in S3 Table. Subsequently, EASE analysis was carried out for each cluster to determine whether certain GO categories are over-represented or not. The over-represented GO categories were selected from each cluster according to their corresponding *P*-value and were shown in Fig 2. A detailed GO annotation of the differentially expressed proteins in each cluster was provided in the S4 Table.

Obviously, cluster 1 was mainly enriched with categories of carbohydrate metabolism, and the proteins in this cluster included 6-phosphogluconate dehydrogenase (PGD), phosphofructokinase (PFK), UDP-glucose 6-dehydrogenase (UGDH), and enolase (ENO1). The proteins in cluster 2 were functionally classified into the categories of nucleosome assembly and organization, including members of histone (H1, H2A, H2B, and H4). A higher proportion of proteins in cluster 3 were associated with tanslatation (i.e. members of eukaryotic translation initiation factors and ribosomal proteins), and energy metabolism (i.e. aldehyde dehydrogenase family 5, acyl-coenzyme A oxidase 3, ATP synthase-coupling factor 6, creatine kinase M-type). Muscle contraction-related proteins such as myosins and alpha-actinin-2, extracellular matrix organization-related proteins such as collagens, were predominantly harbored in cluster 4. Some of the over-represented GO categories along with the differentially expressed proteins were summarized in Table 1.

**Table 1:** Relative expression abundance of the representative proteins in four clusters at the early stage of planarian head regeneration.

Accession	Protein Name	Gene	MW [kDa]	calc. pI	Coverage	Unique Peptides	2h*	6h*
**Carbohydrate and energy metabolism (Cluster 1 and 3)**								
F1R2A3	ATP-dependent 6-phosphofructokinase, platelet type	PFKP	35.8	7.0	8.0	2	**0.52**	1.10
P85968	6-phosphogluconate dehydrogenase, decarboxylating	PGD	53.2	7.0	29.4	8	**0.56**	0.94
P30835	6-phosphofructokinase, liver type	PFKL	85.3	7.4	6.3	2	**0.61**	1.14
D4A7P8	2-oxoglutarate dehydrogenase, mitochondrial	OGDH	115.2	6.9	24.1	10	**0.65**	**1.76**
O70199	UDP-glucose 6-dehydrogenase	UGDH	54.9	7.5	37.9	9	**0.66**	1.05
Q64428	Trifunctional enzyme subunit alpha, mitochondrial	HADHA	82.6	9.1	36.6	20	**0.69**	0.83
P55159	Serum paraoxonase/arylesterase 1	PON1	39.3	5.2	23.9	7	**0.70**	0.81
O02572	Enolase	ENO1	17.1	9.0	37.9	4	**0.70**	0.96
P21213	Histidine ammonia-lyase	HAL	72.2	6.5	20.9	10	**0.71**	0.83
G3V945	Aldehyde dehydrogenase family 5, subfamily A1	ALDH5A1	56.1	8.1	24.9	10	1.32	**0.70**
Q1MTI4	Triosephosphate isomerase A	TPI1A	26.8	5.0	29.8	2	**0.57**	**0.56**
Q90XG0	Triosephosphate isomerase B	TPI1B	26.8	6.9	70.6	2	**1.41**	1.01
F1QXK3	Acyl-coenzyme A oxidase 3	ACOX3	70.1	7.6	9.9	4	**1.41**	1.14
D3ZSC5	Cystathionine beta-synthase	CBS	52.4	6.7	33.5	7	**1.44**	1.01
A9JRE6	Dmgdh protein (Fragment)	DMGDH	97.4	7.0	25.7	3	**1.44**	0.95
B0BNG1	Probable proline dehydrogenase 2	PRODH2	51.1	8.5	21.1	8	**1.46**	0.77
Q5SPG8	Acyl-coenzyme A thioesterase 1	ACOT1	48.6	8.3	9.8	3	**1.47**	1.04
Q68FT3	Pyridine nucleotide-disulfide oxidoreductase domain-containing protein 2	PYROXD2	62.8	8.2	4.5	2	**1.49**	0.93
Q6NZW7	Dihydrolipoamide succinyltransferase	DLST	48.6	8.7	11.2	2	**1.49**	0.98
B1WC61	Acad9 protein	ACAD9	68.8	7.7	23.2	13	**1.50**	1.26
Q6PBX8	NADH dehydrogenase [ubiquinone] flavoprotein 2	NDUFV2	26.6	5.6	13.9	3	**1.52**	1.19
P21571	ATP synthase-coupling factor 6, mitochondrial	ATP5J	12.5	9.4	46.3	6	**1.72**	0.88
Q5BK17	Iodotyrosine dehalogenase 1	IYD	32.8	6.6	7.7	2	**1.83**	1.14
P00564	Creatine kinase M-type	CKM	43.0	7.1	21.0	5	**3.01**	**2.89**
**Nucleosome assembly and organization (Cluster 2)**								
P15865	Histone H1.4	HIST1H1E	22.0	11.1	18.7	3	**0.44**	**0.53**
Q7ZUY3	Histone H2A.x	H2AFX	15.0	10.7	39.4	3	**0.38**	**0.53**
P0C0S7	Histone H2A.Z	H2AFZ	13.5	10.6	31.3	2	**0.68**	0.74
A7E2M8	Histone H2B	HIST2H2BE	13.6	10.4	48.4	2	**0.50**	**0.53**
A3KPR4	Histone H4	HIST1H4A	11.4	11.4	53.4	3	**0.61**	**0.71**
Q6NYV3	Histone H1.0	H1F0	21.2	10.9	9.6	2	**0.24**	**0.28**
B8JN00	Histone H2A	H2AFY2	24.3	10.2	9.8	2	**0.62**	**0.56**
**Translation (Cluster 3)**								
P62755	40S ribosomal protein S6	RPS6	28.7	10.8	24.9	2	**1.41**	0.83
P62083	40S ribosomal protein S7	RPS7	22.1	10.1	44.9	5	**1.66**	0.87
P23358	60S ribomal protein L12	RPL12	17.8	9.4	59.4	5	**1.67**	0.90
H7C5Y5	60S ribosomal protein L6	RPL6	33.6	10.7	29.6	7	**1.38**	**0.61**
Q6P3V8	Eukaryotic translation initiation factor 4A1	EIF4A1	46.1	5.5	37.0	6	**1.58**	1.00
A4V6L7	DEAD box polypeptide 48 protein	EIF4A3	43.1	7.8	7.7	2	**1.65**	1.01
**Protein localization (Cluster 3)**								
F1M6Z1	Apolipoprotein B-100	APOB	509.4	7.2	3.1	11	**1.41**	1.03
Q5PQK5	Radixin	RDX	68.5	6.3	19.0	5	**1.59**	0.91
Q3B7E7	Sorting nexin 3	SNX3	18.8	8.6	12.4	2	**1.80**	0.81
Q4KM74	Vesicle-trafficking protein SEC22b	SEC22B	24.7	8.5	20.5	4	**1.55**	0.92
B2RZD1	Protein Sec61b	SEC61B	10.0	11.6	33.3	2	1.20	**0.61**
**Muscle contraction (Cluster 4)**								
Q76BG8	Fructose-bisphosphate aldolase	ALDOA	36.1	7.9	29.9	8	**1.86**	**2.15**
G3V885	Myosin-6	MYH6	223.4	5.7	30.6	3	1.11	**1.41**
D3ZCV0	Protein Actn2	ACTN2	103.8	5.5	31.0	8	**1.66**	**1.51**
F1M789	Protein Myh13	MYH13	208.8	5.6	21.5	5	**1.67**	**2.36**
F1RBV5	Uncharacterized protein	MYH4	221.6	5.8	26.3	2	**1.47**	**2.22**
B6RK61	Myosin heavy chain 7B	MYH7B	221.5	5.9	9.07	3	**1.45**	1.29
Q7LZ84	Myosin heavy chain, cardiac and skeletal muscle	MYH7	45.2	5.8	47.04	6	**1.45**	1.29
**Extracellular matrix organization (Cluster 4)**								
F1LNH3	Protein Col6a2 (Fragment)	COL6A2	109.4	6.6	2.0	2	**1.70**	**1.62**
Q9W7R9	Alpha1 type II collagen	COL2A1	135.0	7.8	5.2	6	**1.85**	1.38
Q9YIB4	Collagen alpha-1(I) chain	COL1A1	137.5	5.6	21.0	24	**2.37**	1.19
P02454	Collagen alpha-1(I) chain	COL1A1	137.9	5.9	3.4	2	**1.69**	0.94
Q91145	Collagen alpha-1(XII) chain	COL12A1	101.6	5.5	5.9	5	**1.74**	1.35
I3NN31	Collagen type I alpha 2	COL1A2	127.7	8.7	5.7	6	**1.68**	1.25
F1QDL1	Collagen, type I, alpha 3	COL1A1B	136.8	5.5	1.9	2	**1.42**	0.98

*The iTRAQ ratios for 2 h and 6 h using 0 h as control, and the values marked in bold represent up-regulated or down-regulated expression of proteins.

### Modulation of various signal pathways at the early stage of planarian head regeneration

**Fig 3:**
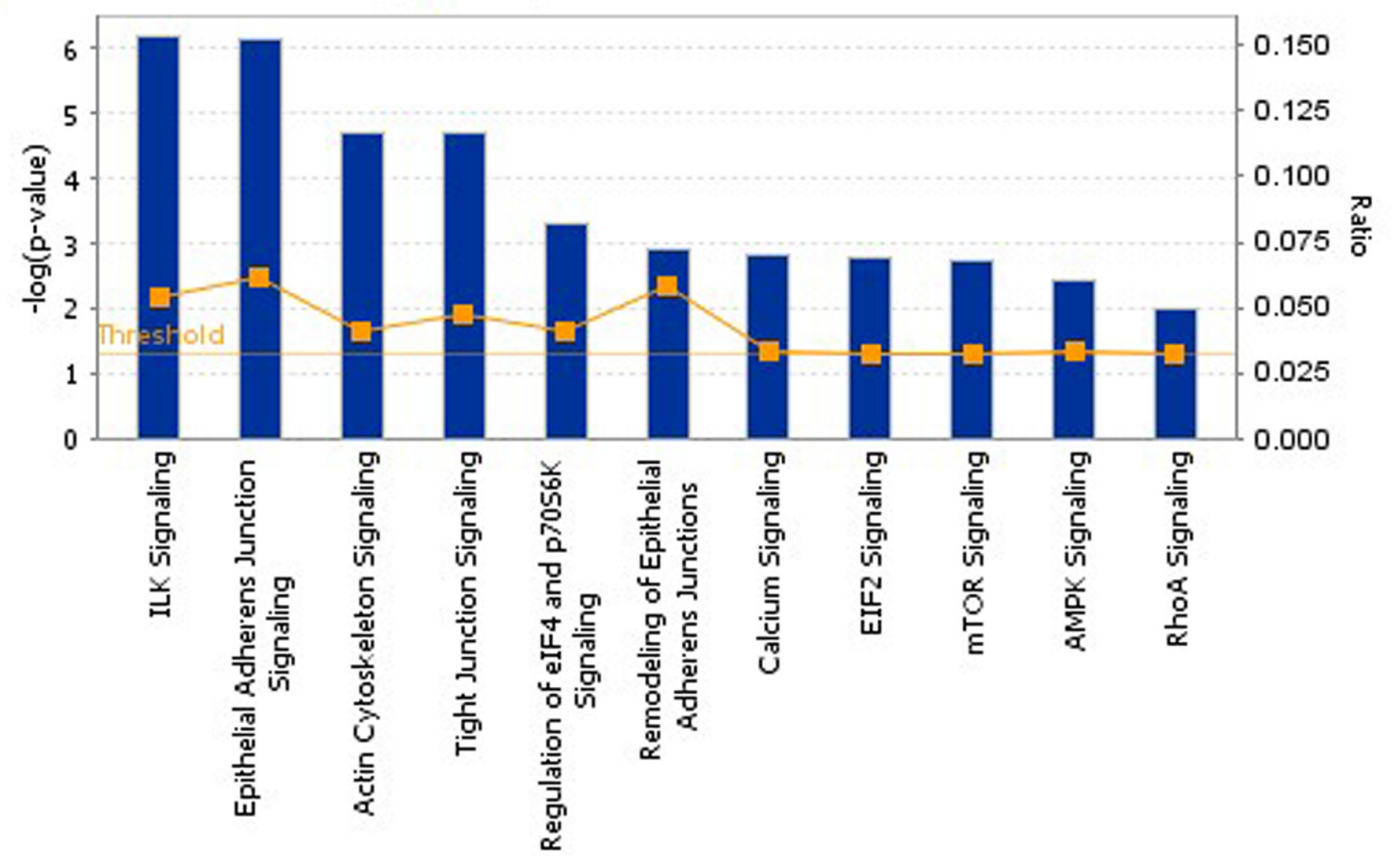
The significantly enriched canonical pathways at the early stage of planarian head regeneration by IPA analysis. Each histogram is a particular canonical pathway. The size of the histogram is correlated with increasing overlap significance (Fisher’s exact test *p*-value).

To further clarify which signaling pathways play important roles at the early stage of PHR, pathway analysis was performed to connect the differentially expressed proteins with canonical biological pathways by IPA software. A Benjamini-Hochberg corrected Fischer's exact test was utilized to calculate the *p*-values associated with a canonical pathway. Additionally, a ratio was used to determine the number of focus molecules to overall molecules in each canonical pathway. Therefore, we analyzed 162 proteins that are differentially expressed at 2 h and 6 h after amputation using IPA, and the significantly enriched canonical pathways are summarized in Fig 3. Among them, “ILK signaling”, “Epithetial Adherens Junction Signaling”, “Actin Cytoskeleton Signaling”, and “Tight Junction Signaling” were highly significantly enriched and involved in cell movement, cell polarity and cell growth. “Regulation of eIF4 and p70S6K Signaling” and “EIF2 Signaling” were highly correlated with protein synthesis through the regulation of translation initiation. “Calcium Signaling” governed the proliferation and differentiation of multipotent stem cells into neuronal fates, and was also found to be regulating planarian regeneration. “mTOR Signaling” plays important roles in regulating cell survival and proliferation. A detailed canonical pathway enrichment of the differentially expressed proteins was provided in S5 Table.

## Discussion

Planarians have amazing ability to regenerate through the activation of pluripotent stem cells, and present an attractive system for molecular investigation of regeneration initiation. Although some researches have been performed on brain regeneration and head regeneration [8, 24, 41, 42], the mechanisms that initiate PHR remain unclear. In this study, we for the first time employed an iTRAQ-based shotgun proteomic approach to study the initiation of PHR. By this high-throughput quantification method, 162 proteins were found to be significantly altered at 2 h and 6 h after amputation. These proteins were functionally related to carbohydrate and energy metabolism, protein synthesis, nucleosome assembly, muscle contraction and wound healing, and involved in various signal pathways including ILK signaling, calcium signaling, EIF2 signaling and mTOR signaling. Below, the most important proteins related to the initiation of PHR are discussed based on their putative biological functions and signal pathways.

### Carbohydrate and energy metabolism

A proteomics study reported that sugar metabolism and energy metabolism was enriched in planarian stem cells, as energy is required for cellular processes including wound healing and regeneration [30]. Consistent with this result, we also found that carbohydrate and energy metabolism was significantly enriched in cluster 1 and 3. Surprisingly, glycolysis was significantly enriched in cluster 1 with the expression pattern of down-regulation at 2 h and slight up-regulation at 6 h after amputation, and the key enzymes for glycolysis including 6-phosphofructokinase (PFK), enolase (ENO1) and triosephosphate isomerase (TPI1) were identified, indicating that glycolysis might be weakened at 2 h, and slightly increased at 6 h of PHR. Our results further support the idea that the amputated tail initially mobilizes the glycogen and lipid reserves during wound healing stage of tail regeneration in lizard [43]. In addition to 2-oxoglutarate dehydrogenase (E1) as one of the key enzymes associated with tricarboxylic acid cycle (TCA cycle) that was down-regulated at 2 h and up-regulated at 6 h after amputation, other key proteins related to TCA cycle and oxidative phosphorylation identified in our study were up-regulated in protein levels, thereby contributing to the maintenance of cellular energy. Furthermore, we found that creatine kinase M-type (CKM) presented a two to three-fold increase in protein levels. CKM, a cytoplasmic enzyme involved in cellular energy homeostasis, reversibly catalyzed the transfer of "energy-rich" phosphate, thus providing the direct energy source for muscle contraction. that TCA cycle and oxidative phosphorylation played important roles for energy supply These results suggested that TCA cycle and oxidative phosphorylation seemed to be preferentially utilized for ATP supply during the first 6 h after amputation while glycolysis appeared to be weakened at 2 h after amputation, which is contrary to the result that glycolysis seemed to be preferentially utilized for ATP supply by surviving hepatocytes in the regenerating livers immediately after partial hepatectomy [44]. The results need to be validated prospectively through other methods.

### Nucleosome assembly

Recent studies reported that the expression level of proteins associated with nucleosome assembly responsible for DNA replication and cell division, was significantly altered in the mitotic regenerative neoblasts that drive the nearly unlimited regenerative power of planarians [42, 45]. In this study, seven histones including H1, H2A, H2B and H4 involved in nucleosome assembly were indentified and grouped into in cluster 2. They exhibited expression patterns of down-regulation at 2 h and 6 h after amputation. More importantly, H2B specifically expressed in planarian neoblasts is likely needed for the progression of neoblasts through the S phase of the cell cycle [46]. We found that H2B was down-regulated at both mRNA and protein level at the early stage of PHR. These results suggested that neoblasts at the wound site had not yet undergone cell division within the first 6 h of PHR, which was confirmed by Wenemoser and Reddien [5] who found that the wound-specific signal that induced the first mitotic peak at 6 h after amputation caused an acceleration of G2 rather than S-phase.

### Translation

Studies have reported that protein synthesis was enhanced during PHR after amputation [42, 47]. In the present study, translation was significantly enriched in cluster 3 whose expression pattern was up-regulated at the early stage of PHR. 40S ribosomal proteins, 60S ribosomal protein and eukaryotic translation initiation factors responsible for translation were identified as significantly changed. More importantly, our result was in agreement with the result of Solana *et al*. [46] that eukaryotic initiation factors important for the initiation of protein synthesis were down-regulated after inhibition of neoblast-inducing planarian regeneration by siRNA experiment. Therefore, we speculate that a certain amount of proteins may be needed for synthesis and assembly of various organelles to facilitate proliferation and growth of neoblasts at the early stage of PHR.

### Muscle contraction facilitated wound closure during planarian head regeneration

Wound closure is facilitated by muscle contraction [10]. A study by Witchley *et al*. [48] reported that muscle tissue is a major source of the positional information that guides tissue turnover and regeneration programs. In the present study, muscle contraction and muscle system process were prominent in cluster 4 by EASE analysis. Meanwhile, we found that muscle contraction-related proteins including myosins and alpha-actinin-2, were up-regulated at 2 h and 6 h after amputation. A recent study of planarian *Schmidtea mediterranea* by Wenemoser *et al*. [8] identified several early wound response genes including myosins, and myosins were induced between 3 and 12 h following amputation and required for proper patterning during regeneration. Another study also found that myosin heavy chain-A was expressed in the pharynx muscles and pharynx-anchoring muscles during the regeneration process of the pharynx in the planarian *Dugesia japonica* [49]. These results suggest that muscle contraction plays important roles in planarian regeneration. In addition, migration of planarian epithelial cells to the wound site is essential for wound closure [50]. We found that collagens (COL1A1, COL1A2, COL2A1, COL6A2, COL12A1) were significantly increased in the protein level at the early stage of PHR, and involved in cell migration, differentiation and regeneration [51–53], indicating that collagens are essential for triggering PHR. These results suggest that muscle contraction and muscle-mediated migration maybe participate in the initiation of PHR by activating the expression of the proteins associated with positional information and muscle contraction.

### Important pathways for priming planarian head regeneration

Studies have indicated that the inhibition of canonical Wnt/β-catenin pathway in planarians promoted head regeneration at the wound site [17, 18, 54]. In the present study, we found that the expression level of β-catenin -1 was decreased in the regenerating head fragments at the mRNA level by qRT-PCR analysis, being agreement with the result that inhibition of β-catenin-1 expression caused planarian to regenerate a head instead of a tail at the wound site. In addition to the canonical Wnt/β-catenin pathway, Ca^2+^ signaling was found to be significantly changed by IPA analysis, being concordant with the findings that Ca^2+^-dependent transcriptional regulatory events are required for PHR [55, 56], suggesting that Ca^2+^ signaling might play an essential role in regulating planarian regeneration. Therefore, we concluded that head regeneration in planarians was promoted by the activation of Ca^2+^ signaling and the inhibition of Wnt/β-catenin pathway.

Furthermore, this study made a discovery that several signaling pathways significantly enriched by IPA analysis were associated with the migration of cells into the wound site, such as ILK signaling, epithetial adherens junction signaling, actin cytoskeleton signaling, and tight junction signaling, indicating that these pathways were likely to participate in regulating the initiation of PHR. Among them, ILK signaling was the most significantly enriched pathway, and ACTN2 and myosins related to this signaling pathway were up-regulated at the early stage of PHR by iTRAQ analysis. Delightedly, studies have found that the activation of ILK after skin wounding is critical for tissue repair and wound healing [22, 57], which confirmed the speculation that ILK signaling might trigger PHR by promoting wound healing. In addition, regulation of eIF4 and EIF2 signaling and EIF2 signaling significantly enriched by IPA analysis were highly correlated with protein synthesis through the regulation of translation initiation. Meanwhile, we also found that eukaryotic translation initiation factors and ribosomal proteins were up-regulated at the early stage of PHR, suggesting that regulation of eIF4 and EIF2 signaling and EIF2 signaling were essential for priming PHR. Recently it has been shown that amputation triggers two peaks in neoblast mitoses during planarian regeneration [5]. The first mitotic peak is a body-wide response to injury and necessary for blastema formation, and this process requires mTOR signaling [58]. In this study, we found that mTOR signaling was significantly enriched at the early stage of PHR. The results further confirmed a key role for mTOR signaling in priming PHR.

## Conclusions

The proteomic technology iTRAQ was employed to analyze changes in the expression of proteins in the initiation of PHR. A total of 162 proteins differentially expressed at 2 h and 6h after amputation were identified. On the basis of GO annotation, these proteins were involved in several functional categories including carbohydrate and energy metabolism, translation, muscle contraction, extracellular matrix organization, etc. Meanwhile, we make a discovery that various signaling pathways, especially ILK, calcium, EIF2 and mTOR signaling, were involved in the initiation of PHR by IPA analysis. In conclusion, we for the first time found that muscle contraction and ILK signaling played important roles in the initiation of PHR through global analysis of proteome dynamics. The findings of this research improve our understanding of the molecular mechanisms underlying head regeneration initiation in planarians.

## Supporting Information

S1 Table Genes and their primer sequences used for qRT-PCR analysis. (XLS)

S2 Table Details for the identified proteins. (XLS)

S3 Table Details for differentially expressed proteins that grouped into four clusters. (XLS)

S4 Table Detailed GO annotations of the differentially expressed proteins. (XLS)

S5 Table Detailed canonical pathways enriched by the differentially expressed proteins. (XLS)
